# Compulsory Removal and Detention of Fever Patients

**Published:** 1899-08-19

**Authors:** 


### COMPULSORY REMOVAL AND DETENTION OF FEVER PATIENTS.

By a Legal Correspondent.

I recently drew attention to a useful decision of the High Court under the Public Health Act, 1875, with regard to the removal of patients to fever hospitals by Justices' order. The construction of S. 124 of that Act is a matter of some difficulty, and has led to a further question being submitted to the determination of the Queen's Bench Division. S. 124 empowers Justices to order the removal to a hospital of any person suffering from a dangerous infectious disorder (of which, by the way, there is no definition in the Act) who is without proper lodging or accommodation. The point now raised is whether such an order can be made where the patient has, as regards himself, proper lodging and accommodation, but cannot be sufficiently isolated so as to prevent the danger of infection to the other inmates in the house. The Justices of Cumberland had refused here to make an order for removal on the ground that there was no sufficient evidence that the patient was "without proper lodging or accommodation" within the meaning of the section, but, having some doubts as to its construction, they stated a case for the opinion of the High Court, so as to have the matter settled. The Court held that S. 124 was directed to the protection of persons from infection, and not merely to the protection of the patient himself, and they remitted the case to the Justices to make an order of removal. There can be no doubt that this decision is a sound one, having regard to the scope and intention of the Public Health Act, which are sufficiently indicated by its title. A contrary ruling would have been little less than disastrous in view of the captious opposition to removal which would certainly be raised in many cases by relatives who would not hesitate to avail themselves of their right to do so. It may be mentioned that S. 12 of the Infectious Diseases (Prevention) Act, 1890, which empowers a local authority to order detention at their expense in a hospital of a patient whose removal from it cannot be safely effected, seems to have avoided the difficulty created by the language of S. 124 of the Public Health Act by expressly providing for his detention in the hospital where he would not on leaving be provided with lodging and accommodation "in which proper precautions could be taken to prevent the spreading of the disorder by such person."

